# Comparative Evaluation of Customized CAD/CAM vs. Stock Titanium Abutments for Immediate Implant Placement in Class II Extraction Sockets: A Randomized Controlled Trial

**DOI:** 10.3390/dj13080371

**Published:** 2025-08-15

**Authors:** Ali Robaian, Mohamed Mofreh Hamed, Yousra Ahmed, Fatma E. A. Hassanein

**Affiliations:** 1 Conservative Dental Sciences Department, College of Dentistry, Prince Sattam bin Abdulaziz University, Al-Kharj 16273, Saudi Arabia; 2 Oral Medicine, Periodontology, and Oral Diagnosis, Faculty of Dentistry, King Salman International University, El Tur 46612, South Sinai, Egypt; mohamed.mofreh@ksiu.edu.eg; 3 Department of Prosthetic Dentistry, Removable Prosthodontic Division, Faculty of Dentistry, King Salman International University, El Tur 46612, South Sinai, Egypt; yousra.elsayed@ksiu.edu.eg

**Keywords:** immediate implants, CAD/CAM abutments, stock abutments, esthetic outcomes, class II extraction sockets, peri-implant soft tissue changes, randomized controlled trial

## Abstract

Background: Immediate implant placement in the esthetic zone, particularly in Class II extraction sockets with partial facial bone loss, presents challenges in achieving soft and hard tissue stability. Customized computer-aided design/computer-aided manufacturing (CAD/CAM) titanium abutments may offer advantages over prefabricated stock abutments. This study compared the clinical, radiographic, and patient-reported outcomes of customized CAD/CAM titanium abutments versus stock Laser-Lok stock abutments. Materials and methods: In a single-center, double-blind randomized clinical trial, 48 patients received immediate maxillary anterior implants restored with either customized CAD/CAM titanium abutments (*n* = 24) or stock titanium abutments (*n* = 24). Primary outcomes included peri-implant probing depth (PD), mucosal height, Pink Esthetic Score (PES), crestal bone level changes, and patient satisfaction assessed at baseline, 6, and 12 months post-loading. Statistical analysis included effect sizes and 95% confidence intervals. Results: At 12 months, the customized abutment group showed significantly shallower PD (mean difference: −0.54 mm; 95% CI: −0.72 to −0.35; *p* < 0.001), higher PES (12.21 ± 0.35 vs. 10.41 ± 1.17; *p* < 0.0001; Cohen’s d = 2.08), and less crestal bone loss (1.75 ± 0.36 mm vs. 2.33 ± 0.52 mm; *p* < 0.0001). Patient satisfaction scores were also higher in the customized group (*p* = 0.003). Within-group improvements were observed in both groups over time. No implant failures occurred. Conclusions: At 1-year follow-up, customized CAD/CAM titanium abutments demonstrated improved peri-implant soft tissue parameters, esthetics, and patient satisfaction compared to stock abutments. While these findings support their use in esthetically demanding immediate implant cases, the short-term duration and single-center design warrant further long-term multicenter studies to confirm durability. Trial registration: Registered at ClinicalTrials.gov on 19/01/2025 (NCT06791655).

## 1. Introduction

Tooth loss in the anterior maxilla has profound esthetic, functional, and psychological consequences. Missing teeth in this visible “esthetic zone” impair speech and chewing and negatively affect facial appearance and self-confidence, collectively reducing quality of life and hindering social interactions. Common causes of anterior tooth loss include periodontal disease, trauma, extensive caries, and developmental anomalies [1,2]. Immediate dental implant placement has become increasingly favored in the esthetic zone, as it preserves alveolar bone by minimizing post-extraction resorption, reduces overall treatment time by eliminating the healing phase, and enhances patient satisfaction by minimizing the edentulous period. However, immediate placement is technically demanding, particularly when the facial (buccal) bone wall is compromised. Without adequate buccal bone, there is a high risk of post-extraction bone resorption and soft tissue recession that can jeopardize the esthetic outcome [3].

Elian et al. (2007) classified extraction sockets by the status of the facial bone wall and soft tissue [4]. Class II sockets are characterized by partial loss of the facial buccal plate, while the gingival soft tissue remains intact. These defects are common in the anterior maxilla and pose particular challenges for immediate implant placement due to the missing buccal bone. Regenerative support is often required to stabilize such sites and achieve an esthetic contour. For example, placing a collagen plug into the socket can stabilize the blood clot, support the soft tissue profile, and protect the area during healing [4], and recent evidence shows that collagen-based biomaterials enhance bone and soft tissue healing around immediate implants [5]. Collagen plugs are biocompatible, easy to handle, and promote hemostasis; by preserving ridge dimensions and soft tissue contours, they counteract the bone resorption and soft tissue collapse that might otherwise compromise esthetics in Class II sites.

A key factor in anterior implant success is the prosthetic abutment design, which shapes the restoration’s emergence profile and supports the surrounding soft tissue. Stock abutments are generic components available in standard sizes, but they often do not optimally conform to an individual’s anatomy. A stock abutment’s shape may provide inadequate gingival support or a suboptimal crown angulation [6,7]. In contrast, customized CAD/CAM abutments are digitally designed and milled for each patient’s unique implant position and tissue contours. Custom abutments allow precise control over the emergence profile, crown position, and soft tissue support [8,9].

Custom abutments are frequently fabricated with titanium due to its excellent biocompatibility and strength. A well-fitting CAD/CAM titanium abutment can be tailored to correct implant angulation and ensure a tight abutment–implant interface. Studies report that custom titanium abutments improve peri-implant soft tissue health and minimize bacterial leakage at the interface [10,11]. Furthermore, advances in digital impression technology have made it easier to use custom abutments: modern intraoral scanners can accurately capture implant positions even in challenging conditions, facilitating precise fabrication [12,13]. These developments support the use of patient-specific abutments for optimal esthetic and functional outcomes in the anterior zone.

Many studies indicate that custom abutments yield superior soft tissue adaptation and esthetic outcomes compared to stock abutments. For example, custom abutments have been associated with more complete papilla fill, stable gingival margins, and higher Pink Esthetic Score (PES) values in the anterior zone [14,15]. These benefits are attributed to the tailored emergence profile and contour of a custom abutment, which guide favorable soft tissue healing.

However, not all evidence favors customization. Pelivan et al. (2023) found no significant differences in soft tissue adaptation or esthetic scores between CAD/CAM custom abutments and stock abutments in anterior implants, suggesting that factors like tissue biotype or clinical protocols might mask the advantages of custom abutments [16]. These mixed findings imply that the benefits of customized abutments may become most apparent in specific, challenging scenarios—underscoring the need for further research in high-risk conditions.

Despite many studies comparing stock and custom abutments, evidence is scarce for immediate implants in compromised anterior sockets. Most comparisons have examined delayed implant placements or well-healed ridges [6,17,18,19], where bone and soft tissues are relatively stable and differences between abutment types may be less pronounced. By contrast, immediate placement in a Class II socket a partial buccal bone defect is a high-risk scenario for tissue recession or contour collapse. To date, no randomized controlled trial has specifically examined stock versus custom abutment performance in this context. Several publications have highlighted the need for research in this situation, calling for studies on immediate implants in sockets with facial bone deficiencies [6,14,15,17]. Therefore, the present study was designed to directly compare customized CAD/CAM titanium abutments versus stock titanium abutments in Class II anterior sockets managed with a collagen plug, to provide evidence-based guidance for this scenario.

Finite element models also support custom titanium abutments: Vautrin et al. (2025) showed a custom-shaped titanium abutment reduced stress in adjacent bone when the buccal plate was deficient [12], and Elleuch et al. (2023) found titanium abutments led to lower crestal bone strain than zirconia [20]. These results suggest that a well-fitting custom titanium abutment can better distribute forces and potentially protect against bone loss.

This trial specifically focuses on immediate implant placement in Class II anterior maxillary sockets (partial buccal plate loss with intact soft tissue) managed with a collagen plug. Such cases carry a high risk of soft tissue recession and facial contour collapse [21,22]. Incorporating a collagen plug is a simple regenerative strategy to stabilize the clot, maintain soft tissue architecture, and preserve ridge dimensions during healing. Hu et al. (2023) reported that placing a collagen sponge between an immediate implant and the socket walls helped maintain crestal bone levels and soft tissue volume [22].

This randomized controlled trial focuses on a challenging scenario: immediate implants in Class II anterior sockets with collagen plug stabilization, comparing custom-designed versus stock abutments. The primary objective is to compare clinical, radiographic, and patient-centered outcomes between implants restored with customized CAD/CAM vs. stock titanium abutments over a 12-month period.

We hypothesized that the use of customized CAD/CAM titanium abutments would result in significantly better outcomes in the above measures compared to the use of stock abutments in the same clinical scenario. Specifically, we anticipated improved soft tissue stability (less recession, higher PES), greater preservation of crestal bone, and higher patient satisfaction in the custom abutment group. The null hypothesis for this study was that there would be no significant differences in clinical, radiographic, or patient-centered outcomes between immediate implants restored with customized CAD/CAM titanium abutments and those restored with prefabricated stock titanium abutments over the 12-month evaluation period. The null hypothesis was that there would be no significant difference in clinical, radiographic, or patient-centered outcomes between the customized CAD/CAM titanium abutments and the stock titanium abutments.

## 2. Materials and Methods

### 2.1. Study Design

This study was a single-center, double-blinded, randomized controlled clinical trial with a parallel-arm design (1:1 allocation). It was conducted in accordance with the Declaration of Helsinki (2013 revision) and CONSORT guidelines. The protocol was approved by the Research Ethics Committee of the Faculty of Dentistry, Tanta University, Egypt (approval code: OMPDR 042113), and registered on ClinicalTrials.gov (NCT06791655).

### 2.2. Sample Size Calculation

A total of 48 patients requiring immediate implants in the anterior esthetic zone (maxillary canine to canine) were enrolled. The sample size (24 per group) was determined using a power analysis to detect a mean difference of 0.5 mm (SD 0.6 mm) with 80% power at α = 0.05. This sample size (*n* = 48) provides >90% power to detect an effect size of 0.8 (two-tailed test). The calculation was informed by data from Patil et al. (2014) [23].

### 2.3. Randomization and Blinding

An independent coordinator performed computer-generated block randomization (www.randomizer.org accessed date: 4th August). Group assignments were concealed in sequentially numbered opaque envelopes. Due to the nature of the intervention, the surgeon was not blinded. However, participants and outcome assessors were blinded to group allocation. All clinical and radiographic evaluations were performed by a single, calibrated examiner uninvolved in treatment delivery. The Pink Esthetic Score (PES) was assessed postoperatively using standardized photographs by two independent, blinded periodontists, with discrepancies resolved by consensus. Since prosthetic designs were standardized across groups, visual differences were minimal, helping to preserve blinding during esthetic evaluation.

### 2.4. Patient Selection

Forty-eight anterior maxillary sites (canine to canine) with remaining roots or non-restorable teeth were selected from an outpatient clinic between December 2022 and October 2023. All sites were confirmed as Type II sockets via cone-beam CT (partial facial bone loss with intact soft tissue). The study purpose was explained, and written informed consent obtained from each patient.

Inclusion criteria: (1) Adults 20–50 years old; (2) a single hopeless or non-restorable tooth without acute infection; (3) sufficient apical and palatal bone for proper implant positioning and primary stability; (4) good oral hygiene and willingness to attend follow-ups; (5) medically healthy individuals.

Exclusion criteria: (1) Medical contraindications to implant surgery; (2) smokers, diabetics, pregnant or lactating women; (3) history of chemotherapy or head/neck radiotherapy; (4) bisphosphonate therapy.

### 2.5. Patients Grouping

Group I (Customized CAD/CAM Titanium Abutment): Twenty-four patients received custom-milled Grade V titanium abutments, fabricated from BioHorizons Ti-6Al-4V ELI (Grade V titanium alloy) discs, conforming to ASTM F136 and ISO 5832-3 standards for surgical implants. The 98.5 mm discs were milled using a 5-axis unit (imes-icore CORiTEC^®^ 350i), and abutments were digitally designed with Exocad Dental CAD software (version 3.1 Rijeka, Exocad GmbH, Darmstadt, Germany). Final polishing was performed mechanically to eliminate micro-roughness and ensure a smooth emergence profile.

Group II (Stock Abutment): Twenty-four patients received prefabricated straight titanium abutments (BioHorizons Laser-Lok^®^ Esthetic Abutments, BioHorizons, Birmingham, AL, USA)., also manufactured from Ti-6Al-4V ELI (Grade V titanium alloy) in accordance with ASTM F136 and ISO 5832-3 standards. These abutments feature a proprietary laser-microtextured collar, engineered with cell-sized microstructures (typically square-wave microgrooves approximately 6–12 µm wide) that promote perpendicular connective tissue attachment and inhibit epithelial downgrowth. These microchannels create a biophysical seal at the soft-tissue margin intended to stabilize peri-implant mucosa and preserve crestal bone.

### 2.6. Surgical and Implant Procedures

**Figure 1:**
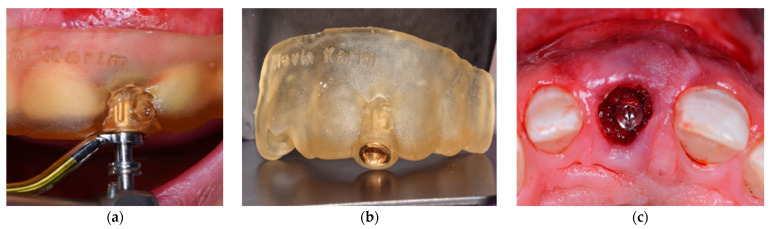
Guided implant placement using a CAD/CAM surgical template. (**a**) Intraoral view showing clinical seating of the 3D-printed surgical guide prior to implant site preparation, confirming passive fit and accurate alignment. (**b**) Occlusal view of the CAD/CAM-fabricated surgical guide with integrated metallic sleeve for precise drill trajectory control. (**c**) Post-implant placement clinical view showing the central incisor region with the implant in situ and initial soft tissue response.

**Figure 2:**
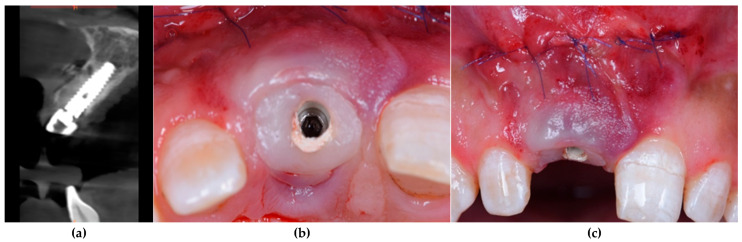
Postoperative assessment of implant placement and soft tissue healing. (**a**) CBCT scan showing accurate 3D positioning of the implant within the alveolar ridge and proximity to adjacent anatomical structures. (**b**) Clinical occlusal view demonstrating optimal emergence profile of the customized healing abutment with early soft tissue adaptation. (**c**) Frontal clinical view showing soft tissue closure and suturing at 2 weeks post-implant placement, highlighting favorable mucosal contour and healing progression.

All patients underwent virtual implant planning with preoperative cone beam computed tomography (CBCT) and intraoral scanning. Stereolithographic surgical guides were fabricated for prosthetically driven implant placement. Tapered internal implants (BioHorizons, Birmingham, AL, USA, pro platform switching design)featuring a deep internal hex conical connection, a Laser-Lok^®^ microtextured collar for soft tissue attachment, and an Resorbable Blast Media (RBM)-treated threaded body to enhance osseointegration were placed equicrestally using a guided surgery kit for precise positioning (Figure 1). Implant diameters (3.8 mm or 4.6 mm) and lengths (12 mm or 13 mm) were selected based on individual site-specific anatomy and bone volume. A standardized resorbable collagen plug (Geistlich CollaPlug^®^) (Bio plug, Biohorizons, Alexandria, AL, USA) was used in all cases to support the facial soft tissue after implant placement. Each plug was trimmed using a sterile scalpel to a uniform cylindrical size (approximately 5 mm length × 3 mm diameter). It was then lightly moistened with sterile saline to enhance adaptability and gently compressed into the facial gap using sterile gauze with mild digital pressure. The plug was not sutured or fixed, and its passive stabilization was maintained by the adjacent socket walls and overlying gingival tissue. The material remained in situ and gradually resorbed over the course of 4 to 6 weeks, consistent with known collagen plug degradation and soft tissue integration patterns [22,24]. Implants were immediately sealed with customized composite healing abutments to preserve the emergence profile (Figure 2).

### 2.7. Abutment Fabrication Workflow

**Figure 3:**
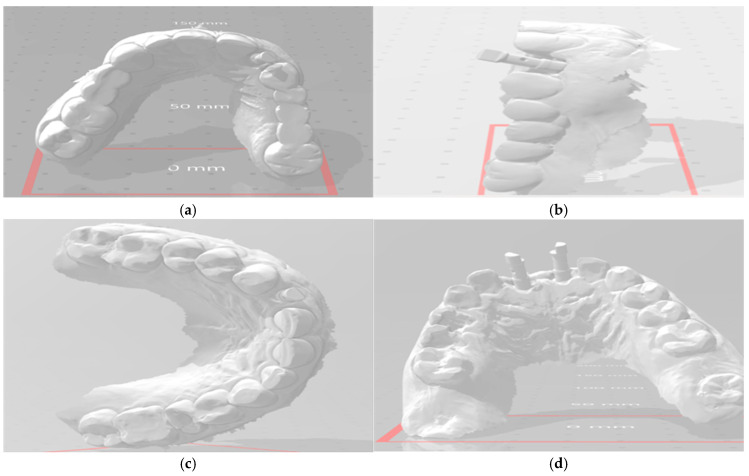
Digital planning and 3D virtual models for guided implant placement. Upper row: (**a**) Occlusal view of the maxillary arch digital scan showing full dentition for surgical planning. (**b**) Lateral view illustrating the virtually planned implant trajectory within the digital model. Lower row: (**c**) Digital model of the mandibular arch in occlusal view. (**d**) Frontal view showing three virtually positioned implants in the anterior maxilla, highlighting spatial alignment and emergence profiles.

**Figure 4:**
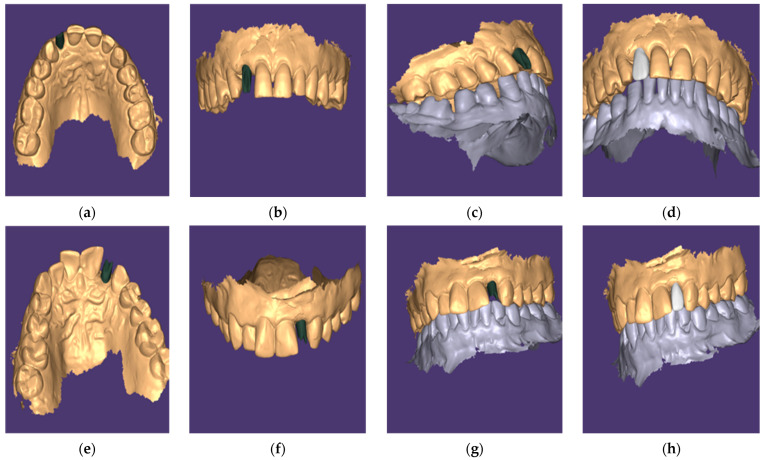
Digital planning workflow showing virtual models of customized (**a**–**d**) and stock (**e**–**h**) abutments. (**a**,**e**): Occlusal views; (**b**,**f**): Frontal views; (**c**,**g**): Buccal views; (**d**,**h**): Final occlusion alignment with opposing arch. Customized abutments demonstrate superior emergence profile and soft tissue support.

Customized Abutments (Group I): After 8 weeks of healing, a scan body (Medentika^®^) was attached to the implant, and a digital impression was taken using a Medit i700 intraoral scanner (Figure 3). All scans were performed by a single calibrated prosthodontist with over 5 years of experience in digital workflows. Operator calibration was ensured prior to patient enrollment through repeated pilot scans and cross-verification against reference STL files. As a result, inter-operator variability was eliminated, and intra-operator reliability was maintained throughout. The scan data were imported into Exocad DentalCAD software (version 3.1 Rijeka, Exocad GmbH, Darmstadt, Germany) to design both the patient-specific titanium abutment and the definitive zirconia crown, respecting the soft tissue emergence profile. The custom abutments were milled from titanium and the crowns from zirconia (VITA YZ ST) using a Roland DWX-52DCi mill (Figure 4). The custom abutments were tried in and polished to a high shine.

Stock Abutments (Group II): After 8 weeks, straight Laser-Lok stock abutments were selected based on implant angulation and soft tissue contours. Crowns were fabricated via conventional protocols (analog or digital impression followed by lab fabrication) for delivery at approximately 10 weeks post-implant.

### 2.8. Standardized Polishing and Cleaning Protocol

Following any adjustments or fabrication, all titanium abutments both customized and stock underwent a standardized polishing and decontamination protocol. Mechanical polishing was first performed using blue (coarse) and pink (fine) silicone polishers (Komet Dental, Germany) in a low-speed handpiece under continuous water irrigation. Final high-gloss polishing was completed with alumina-based DiaShine^®^ polishing paste applied using a felt wheel. This approach has been shown to reduce bacterial adhesion and support soft tissue healing around titanium abutments [25,26]. Subsequently, all abutments were ultrasonically cleaned in 70% isopropyl alcohol for 10 min, rinsed with sterile distilled water, and autoclaved at 134 °C for 20 min before clinical placement. This validated sterilization method effectively removes surface contaminants and preserves biocompatibility, ensuring that surface treatments did not confound soft tissue outcomes in either group [18,27].

### 2.9. Prosthetic and Postoperative Protocol

All patients received comprehensive postoperative care instructions.

Phase I (Preoperative): Full-mouth scaling, oral hygiene reinforcement, and CBCT assessment to classify the socket type.

Phase II (Surgical): Atraumatic tooth extraction under local anesthesia, guided implant placement, collagen plug insertion, and placement of a healing abutment or provisional to seal the implant.

Phase III (Immediate Post-op): Amoxicillin–clavulanate (875/125 mg) was given every 12 h starting one day pre-op and for 5 days post-op. Ibuprofen (600 mg) was prescribed twice daily as needed for pain. Patients rinsed with 0.1% chlorhexidine twice daily for 2 weeks.

Phase IV (Prosthetic): At 8 weeks post-op, patients returned for prosthetic procedures. In Group II, stock abutments were placed and used directly for crown try-in and impression procedures. In Group I, the healing abutment was briefly removed to attach a scan body for digital impression, then reinserted to preserve soft tissue contours. STL files were sent to the lab for custom abutment and zirconia crown fabrication. After 2 weeks, restorations were tried in and delivered.

All final prostheses in both groups were screw-retained zirconia crowns, torqued to 30 N·cm using a calibrated driver. No intraoral cementation was performed, eliminating the risk of excess cement. Oral hygiene instructions were reinforced for all patients.

### 2.10. Outcome Assessments

Photographic Documentation: Standardized digital photographs of the implant sites were taken at crown insertion (baseline, T0) and at follow-ups (T1 = 6 months, T2 = 12 months).

### 2.11. Clinical Assessment

1. Probing Depth (PD): Peri-implant PD was measured using a UNC-15 probe at two aspects (mesial and distal) for each implant. Baseline PD was recorded prior to extraction at the corresponding tooth sites, and implant PD was measured at T1 and T2.
2. Peri-implant Mucosal Level: Changes in soft tissue level were assessed by measuring the height of the mesial papilla, mid-facial mucosa, and distal papilla at T1 and T2 relative to baseline. The average change of these three points represented overall peri-implant mucosal level change.
3. The Pink Esthetic Score (PES): The PES was evaluated at T1 and T2 to rate the peri-implant soft tissue esthetics. Seven PES criteria (mesial/distal papilla fill, soft tissue level, contour, ridge morphology, soft tissue color, and texture) were scored 2 (best) to 0 (worst). The maximum PES is 14, indicating ideal tissue esthetics around the implant crown [28].

### 2.12. Radiographic Assessment

Each patient had CBCT scans at T0 (crown delivery), T1, and T2 to evaluate crestal bone levels. Standardized cross-sectional views were used. A line tangent to the implant’s labial threads was drawn, and a perpendicular line at the implant’s crest was used to measure crestal bone thickness or level at each time point. Changes in crestal bone level between time points were calculated to quantify bone loss.

### 2.13. Patient Satisfaction

Patient-reported outcomes were measured using a visual analog scale (VAS) questionnaire administered at one week after crown insertion (baseline satisfaction, T0) and at T2 (12 months). Patients rated their satisfaction from 0 (completely dissatisfied) to 10 (completely satisfied) for various domains: gum appearance, phonetics/speech, chewing comfort, and overall satisfaction. The questionnaire provided clear explanations of the scale, and both functional (chewing, ease of cleaning) and esthetic (appearance of gums, crown shape/color) aspects were covered [29].

### 2.14. Statistical Analysis

Data were analyzed using *Stata* 18.0 (StataCorp., College Station, TX, USA). The Shapiro–Wilk test assessed normality. Continuous variables are presented as mean ± standard deviation (SD) if normally distributed and as median (interquartile range) for non-normal data. For between-group comparisons, an independent samples *t*-test was used for normally distributed metrics, and the Mann–Whitney *U* test was used for non-parametric data. Within-group changes over time were evaluated with paired *t*-tests or Wilcoxon signed-rank tests as appropriate. Categorical data (e.g., gender, implant site distribution) were compared with chi-square or Fisher’s exact tests. A *p*-value ≤ 0.05 was considered statistically significant. Effect sizes were calculated: Cohen’s d for parametric differences and rank-biserial *r* for non-parametric comparisons to quantify the magnitude of effects.

## 3. Results

**Figure 5:**
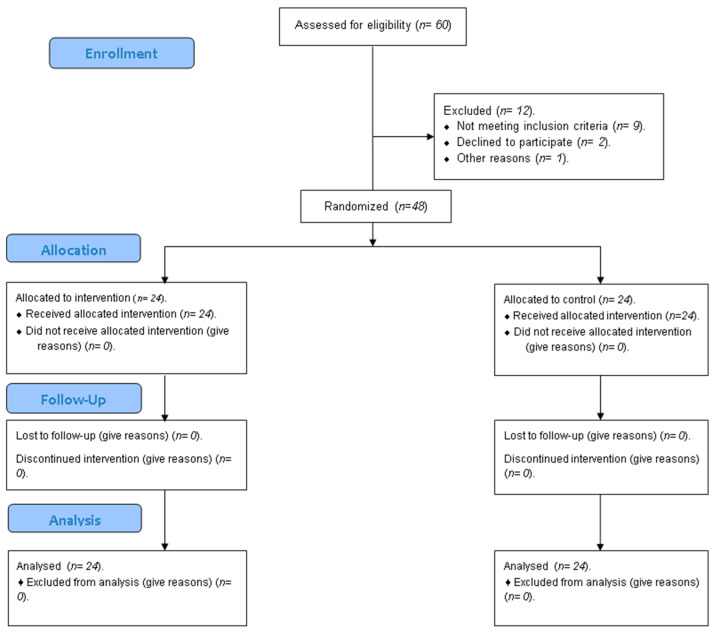
CONSORT flow chart.

Patient Flow: Initially, 60 patients were assessed for eligibility; of these, 12 were excluded. Consequently, a total of 48 patients were enrolled and randomly allocated into two groups (n = 24 per group). All patients received their allocated interventions (customized CAD/CAM abutment or stock abutment). All patients completed the 12-month follow-up period, and data from all 48 patients (24 per group) were included in the final analysis as summarized in Figure 5.

### 3.1. Patients’ Demographic Data

Baseline characteristics of the study groups are detailed in Table 1. There was no statistical difference between the two in terms of age, gender distribution, insertion torque, and implant site.

### 3.2. Clinical Assessment

Probing Depth (PD): Both groups showed a significant reduction in peri-implant probing depths from baseline to follow-ups. At baseline (T0), mean mesial PD did not differ significantly between groups (customized: 3.59 ± 0.55 mm, 95% CI: 3.36–3.82 vs. Stock: 3.39 ± 0.65 mm, 95% CI: 3.09–3.67; p = 0.242; Cohen’s d = 0.34) and distal PD (3.98 ± 0.60 mm, 95% CI: 3.73–4.24 vs. 3.79 ± 0.65 mm, 95% CI: 3.53–4.06; *p* = 0.275; Cohen’s d = 0.31), indicating minimal baseline imbalance. By 6 and 12 months, the customized abutment group had significantly shallower PD than the stock group on both mesial and distal aspects (*p* < 0.0001 at T1 and T2 for both mesial and distal comparisons). The effect sizes at follow-up were large to very large, particularly at 12 months with mesial PD (Cohen’s d = 1.86) and distal PD (Cohen’s d = 3.31), reflecting strong clinical relevance. Within each group, PD values dropped significantly from T0 to T1 and T2 (all *p* < 0.01), reflecting successful peri-implant tissue stabilization. Table 2 presents detailed PD values. Notably, at 12 months the mean mesial PD was 1.75 ± 0.49 mm (95% CI: 1.54–1.95) for customized vs. 2.60 ± 0.41 mm (95% CI: 2.42–2.76) for stock abutments (*p* < 0.0001), and mean distal PD was 1.69 ± 0.49 mm (95% CI: 1.49–1.90) vs. 3.20 ± 0.42 mm (95% CI: 3.02–3.38) (*p* < 0.0001), indicating better maintenance of shallow sulcus depths with customized abutments.

Peri-Implant Mucosal Levels: The average vertical soft tissue changes (combining papillae and mid-facial measurements) were modest in both groups, indicating slight soft tissue gain or minimal recession. However, the between-group differences were statistically significant at both follow-up points (Table 3). At 6 months (T1), the customized abutment group demonstrated a mean soft tissue gain of + 0.645 ± 0.063 mm (95% CI: 0.618–0.672 mm) compared to + 0.606 ± 0.057 mm (95% CI: 0.582–0.630 mm) in the stock group (*p* = 0.031; Cohen’s d = 0.65), indicating a moderate effect size. At 12 months (T2), the difference was more pronounced, with the customized group showing + 0.649 ± 0.054 mm (95% CI: 0.626–0.672 mm) versus + 0.602 ± 0.051 mm (95% CI: 0.580–0.624 mm) for the stock group (*p* = 0.004; Cohen’s d = 0.89), representing a large effect size. Positive values reflect soft tissue height gain relative to the baseline reference. Within-group comparisons from 6 to 12 months revealed no significant differences (*p* > 0.75 in both groups), suggesting that peri-implant mucosal levels stabilized after the initial healing phase.

**Figure 6:**
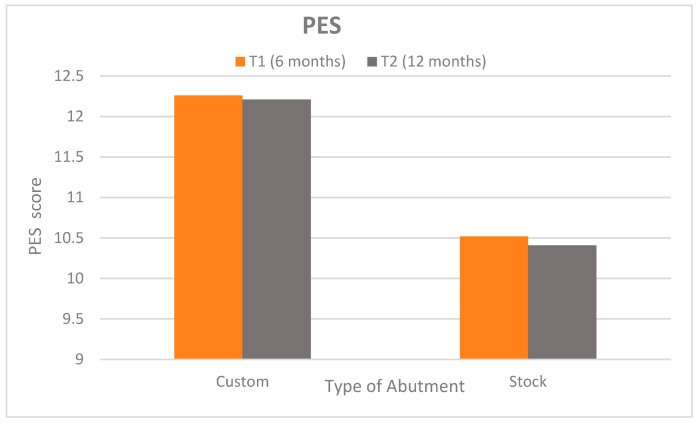
Clustered bar chart shows PES in each group at 6 and 12 months.

Pink Esthetic Score (PES): Peri-implant soft tissue esthetics were superior in the customized abutment group at both follow-ups (Figure 6). At 6 months (T1), the customized group achieved a mean PES of 12.26 ± 0.38 (95% CI: 12.10–12.42) versus 10.52 ± 0.89 (95% CI: 10.14–10.90) for the stock group (*p* < 0.0001; Cohen’s d = 2.54), indicating a very large effect size. At 12 months (T2), the scores were 12.21 ± 0.35 (95% CI: 12.06–12.36) vs. 10.41 ± 1.17 (95% CI: 9.92–10.90), respectively (*p* < 0.0001; Cohen’s d = 2.08), again reflecting a very large difference. These findings suggest clinically and esthetically meaningful superiority of customized abutments in preserving peri-implant esthetics. Within-group PES comparisons from T1 to T2 were non-significant (*p* = 0.433 and *p* = 0.697), indicating stable esthetic outcomes over time.

### 3.3. Radiographic Assessment

**Figure 7:**
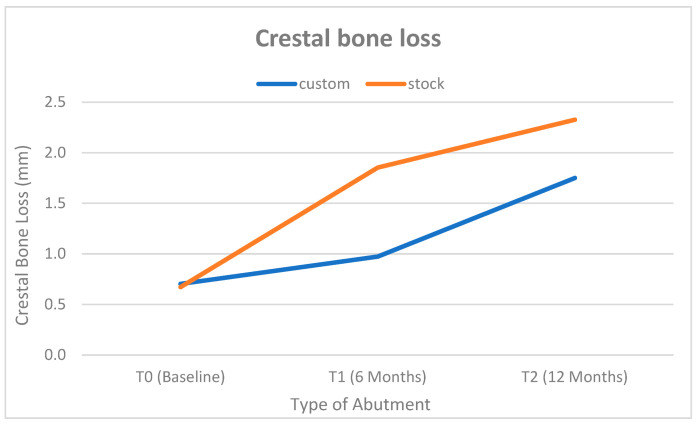
Linear chart shows level of crestal bone loss in each group at three time-points.

Bone loss was significantly lower in the customized abutment group. At baseline (post-loading T0), mean crestal bone levels were comparable between groups (customized: 0.703 ± 0.475 mm; 95% CI: 0.50–0.90 vs. Stock: 0.672 ± 0.032 mm; 95% CI: 0.66–0.69; *p* = 0.751; Cohen’s d = 0.09), indicating negligible baseline differences. By 6 months (T1), the stock abutment group showed significantly greater bone loss (1.852 ± 0.232 mm; 95% CI: 1.75–1.95) compared to the customized group (0.972 ± 0.755 mm; 95% CI: 0.65–1.29; *p* < 0.0001; Cohen’s d = 1.58), reflecting a large effect size. This trend persisted at 12 months (T2), with cumulative bone loss reaching 2.327 ± 0.524 mm; 95% CI: 2.11–2.55 in the stock group versus 1.750 ± 0.363 mm; 95% CI: 1.60–1.90 in the customized group (*p* < 0.0001; Cohen’s d = 1.28), further supporting the bone-preserving benefit of customized CAD/CAM abutments. Figure 7 illustrates these differences. Within-group analyses confirmed significant progressive bone loss from 6 to 12 months in both groups (additional loss between T1 and T2, both *p* < 0.0001), although the rate of loss appeared to slow in the custom group after 6 months.

### 3.4. Patient Satisfaction

Patient-reported outcomes favored the customized abutment group in most categories (Table 4).

Gum Appearance: At baseline (1 week post-restoration), patients with customized abutments rated gum esthetics highly (median VAS = 9, mean ~8.95) versus a lower median of 6 in the stock group. At 12 months, the customized group’s gum appearance scores remained high (median 10, mean ~9.20), while the stock group’s remained lower (median 7, mean ~6.7). These differences were statistically significant at both time points (*p* < 0.001), with very large effect sizes (Cohen’s d ~2.9–3.0). There were no significant changes within each group from baseline to 12 months for gum appearance (*p* = 0.16 custom, *p* = 0.6 stock), indicating patients’ esthetic perceptions remained consistent.

Phonetics (Speech): Both groups reported excellent speech outcomes. Median scores were 9–10 at both baseline and 12 months in both groups. No significant between-group differences were found (*p* = 0.4), and scores did not change over time (in fact, many patients gave identical scores at both times, so no within-group comparison was computed as the variable was constant).

Chewing Comfort: Customized abutment patients reported significantly better chewing comfort than stock abutment patients at both baseline and 12 months. At baseline, median VAS for chewing was 9 in the custom group vs. 7 in the stock group (*p* = 0.005, *d* = 1.3). These scores remained the same at 12 months (median 9 vs. 7, *p* = 0.005, *d* = 1.3). Within-group changes in chewing comfort were negligible (most patients reported consistently high or consistently moderate comfort; “Not computed” in Table 4 because the values were unchanged for many patients).

Overall Satisfaction: Patients with customized abutments were more satisfied overall with their implant treatment. At baseline, the customized group’s overall satisfaction median was 9 (range 8–10) versus 7 (range 6–8) in the stock group (*p* < 0.001, *d* ≈ 2.36). At 12 months, medians were 9 vs. 7 again (*p* < 0.001, similarly large effect). There were no significant within-group changes in overall satisfaction (custom *p* = 0.23, stock *p* = 0.6), indicating that the initial impressions of overall success persisted through 1 year.

## 4. Discussion

This study demonstrated that customized CAD/CAM titanium abutments led to significantly better clinical and esthetic outcomes compared to stock abutments, including shallower probing depths, enhanced soft tissue height, higher Pink Esthetic Score (PES), and greater patient satisfaction. Based on these findings, the null hypothesis is rejected. Statistically significant differences were observed between the customized and stock abutment groups in terms of peri-implant soft tissue profile and patient-related outcomes. The early formation and long-term stability of peri-implant tissues both soft and hard are essential to implant success. Numerous factors, including soft tissue volume and quality, surgical technique, and prosthetic and abutment design, significantly influence this stability [30,31].

Precision and fit of the interface between the implant and abutment are essential for long-term clinical success. An accurately adapted interface improves mechanical stability as well as decreases biological complications. Notably, abutment material selection is correlated with soft tissue health as well as crestal bone preservation [32,33]. Customized healing abutments have been reported to improve immediate gingival contouring, decrease second-stage surgery requirements, as well as minimize the risk of premature loading [34]. Based on such benefits, the current study examined the effect of customized CAD/CAM titanium abutments compared with stock abutments on Class II extraction ridge hard and soft tissue alterations employing a collagen plug. To the best of our knowledge, this is the first clinical study to evaluate the esthetic and biological performance of customized CAD/CAM titanium abutments in immediate implant placement within Class II extraction sockets managed with collagen plugs.

Long-term crestal bone height maintenance around osseointegrated implants is frequently utilized as a key criterion for evaluating the success of various implant systems. It has been proposed that crestal bone loss of 1.5 mm or less during the first year of functional loading, and 0.2 mm or less per year thereafter, serves as a benchmark for successful implant-prosthodontic treatment [35]. However, it is important to note that radiographic evidence of bone contact with an implant does not necessarily indicate osseointegration at the histological level. Computer-aided image analysis has demonstrated higher diagnostic accuracy and greater sensitivity for detecting minimal changes in periodontal tissue [36]. Peri-implant mucosa and implant surfaces complicate direct comparisons of probing depth measurements between teeth and implants. Values of periodontal pocket depth must also be interpreted in the context of the surgical positioning of implants, as a progressive increase in probing depth is an alarming sign [37].

Regarding peri-implant soft tissues, our study demonstrated that both custom and stock abutments resulted in significant improvements in mesial and distal probing depth (PD) from baseline to six months, with continued progress up to 12 months, after which the conditions tended to stabilize. These findings may be attributed to the effectiveness of initial periodontal treatments and post-implant adaptations. The notable reductions in PD in both groups emphasize the efficacy of periodontal management strategies, particularly in the early post-implantation phase, and underscore the importance of maintaining these improvements over time [38].

However, the custom abutment group exhibited greater improvements in PD measurements compared to the stock abutment group. This aligns with the findings of Valsan et al., who reported that customized CAD/CAM abutments provide an excellent finishing line, eliminating sharp edges, compensating for poor implant angulation, and offering a biological advantage by supporting and interacting with soft tissues [39]. Conversely, Pelivan et al. found no significant difference in sulcus-probing depth between patients with stock and custom abutments. This discrepancy could be attributed to differences in study design, implant manufacturing techniques, and abutment fabrication methods [16].

Regarding soft tissue height, the measurements in this study confirmed that custom CAD/CAM abutments resulted in greater soft tissue height gain compared to stock abutments. These findings indicate that custom abutments may offer superior long-term outcomes in soft tissue management. This aligns with the conclusions of Lopes et al., who reported that CAD/CAM abutment-supported restorations in titanium and zirconia exhibited a lower mean papillary recession index than stock abutments, thereby enhancing papilla support and preventing excessive papilla compression [40]. Similarly, our results align with the retrospective study by Menchini-Fabris et al. (2023), which showed that customized healing abutments contributed to improved tissue support and stable peri-implant mucosa in periodontally compromised extraction sites over a 1-year follow-up period [41]. For instance, Pelivan et al. (2022) observed no statistically significant difference in soft tissue parameters between CAD/CAM and stock abutments over a similar period [16]. These contrasting findings suggest that clinical context, including patient-specific anatomy, implant positioning, surgical technique, and operator experience, may significantly influence abutment performance [20].

Our results can be attributed to CAD/CAM abutments providing a predictable fit and durability. They also allow for the modification of all prosthetic parameters including the emergence profile, thickness, finish line position, and outer contour. Consequently, CAD/CAM abutments can improve papilla support and prevent excessive papilla compression [6,42].

Regarding the results of the Pink Esthetic Score (PES), it was shown that there were statistically significant differences between custom and stock abutments, with custom abutments achieving higher PES than stock abutments. The 1.8-point difference in PES observed between groups exceeds the clinically perceptible threshold described in previous literature, reinforcing the visual and biological advantages of customized abutments. This finding was in agreement with Valsan et al., who explained that CAD/CAM abutments have an excellent finishing line, thus avoiding sharp edges. They also compensate for poor implant angulation and provide a biological advantage, as they support and interact with the soft tissues, unlike stock abutments, where it is the crown that performs this function [39]. Although our protocol did not include immediate provisionalization, the volumetric soft tissue assessment by Menchini-Fabris et al. (2023) using intraoral scans showed that immediate restorations help preserve the external gingival contour, supporting our PES findings by emphasizing the role of abutment design in tissue shaping and long-term esthetic maintenance [43].

The enhanced clinical outcomes observed with customized CAD/CAM titanium abutments are attributable to superior mechanical strength, precision fit, and optimized surface finish. Compared to zirconia, titanium abutments exhibit higher fracture resistance and long-term durability, especially under functional loading, making them more reliable in posterior or high-stress sites [44,45]. Although zirconia offers esthetic benefits, it may be prone to mechanical complications over time [46]. CAD/CAM abutments improve soft tissue outcomes by enabling customized emergence profiles that reduce mucosal compression and better support gingival architecture [47]. Moreover, studies show that smoother abutment surfaces achieved using CAD/CAM precision or plasma treatments enhance soft tissue adhesion and reduce biofilm formation, leading to improved peri-implant health compared to conventional machined stock abutments [25,26]. These biological and mechanical advantages validate the clinical utility of CAD/CAM titanium abutments over their stock or zirconia counterparts.

Regarding radiographic measurement of the crestal bone loss, the results of this study showed that distinct patterns in bone level changes within each abutment type. Custom abutments show significant long-term changes, especially from 0 to 12 months and between 6 to 12 months. This was in accordance with Pelivn et al. who revealed that total crestal bone loss between initial and control measurements after twelve months showed no significant differences. However, total bone gain after one year was slightly higher in subjects with custom abutments compared to those with stock abutments, though the difference was not statistically significant. This may be attributed to the design and fabrication of the abutment surface significantly affect the fit between the abutment and the implant that affect the stress transmitted to implant and implant bone interface [16].

Patient satisfaction mirrored clinical outcomes: customized CAD/CAM abutments demonstrated superior performance compared to stock abutments, particularly in terms of gum appearance and chewing comfort. This may be attributed to the customization of abutment parameters in implant rehabilitation, which enhances soft tissue support and improves the papillary recession index. However, it has been reported that these outcomes are influenced by mucosal thickness, which affects peri-implant soft tissue regardless of the abutment material used. Furthermore, proper soft tissue management during implant placement is essential, with a focus on preserving an adequate amount of keratinized mucosa at the facial aspect [48,49].

Customized CAD/CAM titanium abutments are especially advantageous in the esthetic zone, where durability and visual harmony are paramount. Their precise fit reduces bacterial infiltration and enhances mechanical integrity. Moreover, titanium’s superior mechanical strength makes it a dependable option for sites with lower bone density or high occlusal loads. In contrast, zirconia abutments though favored for their esthetic benefits in thin biotypes due to their tooth-colored hue have shown certain biomechanical limitations. While zirconia reduces mucosal discoloration and may enhance esthetic integration, several studies report higher incidences of chipping and framework fractures in long-term clinical use [50]. A comprehensive review by Halim et al. (2022), which analyzed eleven systematic reviews, concluded that titanium abutments offer better mechanical resistance and fewer technical complications such as fractures or chipping, despite equivalent soft tissue responses between materials [50]. Moreover, a recent systematic overview by Laleman et al. (2023) found no significant difference in marginal bone level or probing depth between titanium and zirconia abutments over a five-year period, although ceramic fractures were reported more frequently in zirconia groups [51]. These findings suggest that, while zirconia may offer esthetic appeal especially in thin mucosal biotypes titanium CAD/CAM abutments deliver superior mechanical reliability, making them well-suited for challenging anterior cases such as immediate placement in Class II sockets managed with collagen plugs.

These findings emphasize the clinical utility of customized abutments in managing complex anterior esthetic cases, particularly where socket preservation with biomaterials like collagen plugs is critical. The use of collagen plugs to stabilize the facial soft tissue in Class II sockets is supported by the recent systematic review by Ahamed et al. (2025), which emphasizes the clinical relevance and biological compatibility of collagen-based biomaterials in the management of peri-implant defect [5]. Future trials should explore histologic responses and long-term soft tissue dimensional stability to validate the biological advantages observed radiographically and clinically.

## 5. Limitation

Despite encouraging results, this study has limitations. The sample size, though informed by power analysis, remains modest and may limit detection of rare events or subtle differences. The one-year follow-up precludes assessment of long-term outcomes. Only titanium abutments were tested; comparisons with zirconia could offer additional insights. All procedures were performed by a single surgical team in a university setting, limiting generalizability. While outcome assessors and participants were blinded, the surgeon was not, and prosthetic-phase visual cues may have introduced bias. Although outcome measures were standardized, the lack of formal inter- and intra-examiner calibration and absence of histological validation restrict the depth of interpretation regarding soft tissue integration. No implant failures or biological/mechanical complications (e.g., peri-implantitis, screw loosening, or abutment fracture) were observed in either group during the 12-month follow-up.

## 6. Conclusions

Within the limitations of this 1-year randomized controlled trial, customized CAD/CAM titanium abutments showed favorable clinical, radiographic, and patient-reported outcomes compared to stock abutments in immediate implants placed in Class II anterior sockets. These findings suggest potential advantages in peri-implant tissue preservation and esthetics when using custom abutments alongside collagen plug techniques. However, larger and longer-term studies are needed to confirm these results and to assess the influence of abutment material versus design.

## Figures and Tables

**Table 1:** Frequencies (n), percentages (%), mean, and standard deviation (SD) values for patient demographics in the two groups.

Characteristic	CAD-CAM Titanium Milled Abutments	Stock Abutment	*p*-Value
**Gender**			0.041 ns
Male	11 (46%)	12 (50%)	
Female	13 (54%)	12 (50%)	
Age in years	30 ± 6.2	28.4 ± 7.5	0.338 ns
Site			0.582 ns
Central incisor	8 (33%)	7 (29%)	
Lateral incisor	10 (42%)	8 (33%)	
Canine	6 (25%)	9 (38)	
Insertion torque in N/cm	46.25 ± 6.3	45.5 ± 7.3	0.685 ns

Values are presented as mean ± standard deviation (SD) for continuous variables and as number (percentage) for categorical variables. The *p*-values indicate between-group comparisons using the independent *t*-test (for age and insertion torque) or chi-square test (for gender and site). “ns” denotes non-significant difference (*p* > 0.05).

**Table 2:** Probing depth (PD) at mesial and distal implant aspects for customized vs. stock abutments. Values are mean PD (± SD) in millimeters. Within-group *p*-values refer to changes from baseline (T0) to 12 months (T2).

Parameter	Customized	Customized	Stock	Stock	*p*-Value	Cohen’s d
	Mean ± SD	95% CI	Mean ± SD	95% CI		
Mesial PD—T0	3.589 mm ± 0.552 mm	3.356–3.822	4.118 mm ± 0.591 mm	3.868–4.367	0.242 ns	0.34
Mesial PD—T1	1.790 mm ± 0.430 mm	1.609–1.972	2.542 mm ± 0.525 mm	2.320–2.763	<0.0001 *	1.56
Mesial PD—T2	1.745 mm ± 0.489 mm	1.538–1.951	2.592 mm ± 0.408 mm	2.420–2.764	<0.0001 *	1.86
Distal PD—T0	3.985 mm ± 0.605 mm	3.729–4.240	3.794 mm ± 0.625 mm	3.530–4.058	0.275 ns	0.31
Distal PD—T1	1.726 mm ± 0.409 mm	1.553–1.899	3.299 mm ± 0.472 mm	3.099–3.498	<0.0001 *	3.56
Distal PD—T2	1.691 mm ± 0.487 mm	1.485–1.896	3.202 mm ± 0.424 mm	3.023–3.381	<0.0001 *	3.31

PD: Probing depth. Values are presented as mean ± standard deviation (SD). T0 = baseline, T1 = 6 months, T2 = 12 months. *p*-values indicate between-group comparisons using the independent samples *t*-test. Cohen’s d represents the effect size. Within-group *p*-values compare changes from baseline (T0) to subsequent time points using paired *t*-tests. “ns” denotes non-significant difference (*p* > 0.05); * indicates statistically significant difference (*p* ≤ 0.05).

**Table 3:** Average peri-implant mucosal level changes (mm) from baseline for customized vs. stock abutments (positive values indicate a coronal gain in soft tissue height). Values are mean change ± SD. Within-group *p*-values compare change from T1 to T2.

Time	Custom		Stock		*p*-Value	Cohen’s d
	(Mean ± SD)	95% CI	(Mean ± SD)			
T1	0.645 mm ± 0.063 mm	0.618–0.672 mm	0.606 mm ± 0.057 mm	0.582–0.630 mm	0.031 *	0.65
T2	0.649 mm ± 0.054 mm	0.626–0.672 mm	0.602 mm ± 0.051 mm	0.580–0.624 mm	0.004 *	0.89

Values represent mean change ± standard deviation (SD) in peri-implant mucosal (soft tissue) level relative to baseline. T1 = 6 months, T2 = 12 months. *p*-values reflect between-group comparisons (independent samples *t*-test) and within-group comparisons from T1 to T2 (paired *t*-test). Cohen’s d indicates effect size. “ns” = non-significant (*p* > 0.05); * indicates statistically significant difference (*p* ≤ 0.05). Positive values reflect a coronal (upward) gain in soft tissue height relative to baseline.

**Table 4:** Patient satisfaction outcomes (VAS 0–10) for gum appearance, phonetics, chewing comfort, and overall satisfaction in each group. Data shown as median (range) and mean (SD). Between-group *p*-values by Mann–Whitney *U* test; within-group changes by Wilcoxon test. Effect sizes: *d* (Cohen’s d for between-group at each time) and *w* (rank-biserial for within-group change).

Variable	Time	Custom Abutment (*n* = 22)		Stock Abutment (*n* = 22)		*p*-Value	Effect Size (d)
		Median (Range)	Mean (SD)	Median (Range)	Mean (SD)		
**Gum appearance**	Baseline	9 (8, 10)	8.95 (0.55)	6 (5, 7)	6.2 (0.85)	<0.001 *	2.9
	12 months	10 (9, 10)	9.20 (0.50)	7 (6, 7)	6.7 (0.90)	<0.001 *	3
	*p*-value	0.16 ns		0.6 ns			
	Effect size (w)	0.943 ns		0.353 ns			
**Phonetics**	Baseline	9 (8, 10)	8.95 (0.55)	10 (9, 11)	9.75 (0.52)	0.4 ns	0.3
	12 months	9 (8, 10)	8.95 (0.55)	10 (9, 11)	9.75 (0.52)	0.4 ns	0.3
	*p*-value	Not computed because the variable is constant		Not computed because the variable is constant			
	Effect size (w)						
**Chewing comfort**	Baseline	9 (8, 10)	8.82 (0.4)	7 (6, 8)	7.10 (1.1)	0.005 *	1.3
	12 months	9 (8, 10)	8.82 (0.4)	7 (6, 8)	7.10 (1.1)	0.005 *	1.3
	*p*-value	Not computed because the variable is constant		Not computed because the variable is constant			
	Effect size (w)						
**Overall satisfaction**	Baseline	9 (8, 10)	8.25 (0.2)	7 (6, 8)	7.05 (0.25)	<0.001 *	2.361
	12 months	9 (8, 10)	8.5 (0.24)	7 (6, 8)	7.25 (0.26)	<0.001 *	2.361
	*p*-value	0.23 ns		0.6 ns			
	Effect size (w)	0.73 ns		0.4 ns			

Values are presented as median (range) and mean ± standard deviation (SD). *p*-values indicate between-group comparisons using the Mann–Whitney U test. Within-group *p*-values and effect sizes (w: rank-biserial correlation) compare changes over time using the Wilcoxon test. Effect size (d) = Cohen’s d. “ns” = non-significant (*p* > 0.05); * indicates statistically significant difference (*p* ≤ 0.05). The phonetics and chewing comfort variables did not vary within groups across time points, so within-group tests were not computed.

## Data Availability

Research data supporting this publication are available from the corresponding author upon request.
